# Interdental brushing behaviour in adults: Influence of periodontal status and socioeconomic factors

**DOI:** 10.1007/s00784-026-07129-7

**Published:** 2026-09-16

**Authors:** Pasquale Santamaria, Francesco Fanelli, Rohan Mangalpara, Thamara Kumar, Sarah Van Der Lith, Giuseppe Troiano, Luigi Nibali

**Affiliations:** 1 Periodontology Unit, Centre for Host Microbiome Interactions, Faculty of Dentistry, Oral & Craniofacial Sciences, King’s College London, London, UK; 2 Department of Clinical and Experimental Medicine, University of Foggia, Foggia, Italy

**Keywords:** Periodontitis, Health behaviour, Social factors, Smoking

## Abstract

**Aim:**

To evaluate patterns of interdental brush (IB) use across different stages and grades of periodontitis and to examine associations with socioeconomic, demographic, and behavioural factors.

**Methods:**

This cross-sectional study included 375 adults referred for secondary periodontal care at Guy’s and St Thomas’ Hospital (London, UK). Periodontal status was classified using the 2018 periodontal classification. Participants completed a 10-item questionnaire on IB use and perceived barriers. Area-level household income was estimated using postcode-linked national statistics. Associations were analysed using chi-squared tests and multivariable logistic and ordinal regression models.

**Results:**

Daily IB use (63.0%) was significantly associated with periodontitis stage (p<0.001) but not grade. Patients with Stage III-IV periodontitis had higher odds of daily IB use (OR=3.11, 95% CI 1.88–5.15, p<.001). Higher income and older age were positively associated with IB use, while current smoking was a strong negative predictor (OR=0.31, 95% CI 0.15–0.67). Common barriers included pain, discomfort, and cost.

**Conclusions:**

A distinct profile of periodontal patients who are less likely to use IBs regularly was characterised by early-stage disease, younger age, lower income, and current smoking status. Dissatisfaction driven by practical, financial, and environmental concerns highlights the need for tailored preventive advice and personalised interdental cleaning strategies to improve adherence.

**Supplementary Information:**

The online version contains supplementary material available at https://doi.org/10.1007/s00784-026-07129-7.

## Introduction

In dentistry, interdental cleaning represents a critical element of self-care, since interproximal areas are highly susceptible for the formation of dental plaque. Contemporary periodontal therapy considers effective self-performed plaque control as a cornerstone of treatment. A randomized clinical trial demonstrated that a prolonged phase of rigorous oral hygiene alone significantly reduced plaque accumulation, bleeding on probing, and probing pocket depth before any subgingival instrumentation, highlighting the substantial therapeutic effect of plaque control itself [1]. It is widely recognised that toothbrushing can leave almost half of dental plaque in the oral cavity [2], therefore interdental cleaning can help in efficiently eliminate dental plaque prevent inflammation of periodontal tissues [3].

Generally, Interdental cleaning is recommended as part of professional oral hygiene instruction during Step 1 periodontal therapy in contemporary S3 periodontal treatment guidelines, reflecting its central role in achieving adequate supragingival plaque control. interdental brushing can be defined as a health behaviour consisting of an action performed in response to external events [4]. However, adherence to performing interdental cleaning, defined as the extent to which a person’s behaviours correspond to recommendations agreed with a healthcare provider, remains low [5]. UK Population data indicate that one in three adults has never flossed or cleaned interdentally, despite these practices being essential for the prevention and management of periodontitis [6]. Behavioural engagement with interdental cleaning is influenced by a range of barriers. At the individual level, common obstacles include limited time, poor access to facilities, financial cost, dental anxiety, health inequalities, and concerns about side effects [7]. These factors reflect the broader social and environmental context in which oral health behaviours occur. Importantly, challenges also exist within the dental profession. Keyworth et al., [8] highlight several barriers faced by dental teams, including a perceived lack of prioritisation of behaviour change, a clinical focus on managing disease rather than promoting preventive behaviours, limited financial incentives for delivering behavioural interventions, a reliance on knowledge-based advice, and uncertainty around professional roles, outcomes, and behaviour-change theory. Understanding why patients do or do-not adopt interdental brushing requires attention to these multilevel influences. The COM-B model (Capability, Opportunity, Motivation, Behaviour) provides a comprehensive framework for behavioural diagnosis [9]. Within COM-B, behaviour occurs only when individuals have the capability (physical and psychological), opportunity (physical and social), and motivation (reflective and automatic) required to enact it. Opportunity is particularly relevant for dental hygiene behaviours: physical opportunity relates to time, money, and access to appropriate tools, while social opportunity encompasses the support, routines, and expectations shaped by families, peers, and wider social networks.

Applying this framework, barriers to interdental cleaning can arise from difficulties with physical capability (e.g., discomfort or difficulty using brushes), psychological capability (e.g., lack of confidence or knowledge), physical opportunity (e.g., cost or availability of brushes), social opportunity (e.g., absence of reminders or encouragement), as well as motivational factors such as perceived importance, habits, and forgetting.

Despite the centrality of interdental cleaning to periodontal care, relatively little is known about how social opportunity shapes interdental brushing among patients with different stages of periodontitis. Given that periodontal patients require consistent interdental cleaning to reverse inflammation and prevent progression, identifying the social and environmental factors that support or hinder this behaviour is clinically essential.

Therefore, the primary aim of this study was to evaluate interdental brush (IB) use among patients with different stages and grades of periodontitis and, secondarily, to assess its relationship with socioeconomic, demographic, and behavioural factors. It was hypothesised that regular daily IB use would be more common among patients with more advanced periodontitis and would be positively associated with favourable socioeconomic characteristics and health-related behaviours, including higher household income, older age, and non-smoking status.

## Materials and methods

A structured self-administered questionnaire was specifically developed for this exploratory study to assess participants’ awareness, usage patterns, perceptions and barriers related to IB use. The questionnaire was designed by the research team, comprising specialist periodontists and researchers with expertise in clinical periodontology and behavioural aspects of oral health. Questionnaire items were informed by the existing literature on oral hygiene behaviours and common barriers to interdental cleaning, together with the authors’ clinical experience in managing patients attending secondary periodontal care.

The questionnaire consisted of ten predominantly closed-ended questions and one optional open-ended question to capture qualitative feedback. Particular emphasis was placed on producing a brief and easily understandable instrument that could be completed within approximately five minutes during the clinical visit. As this represented an exploratory observational study, the questionnaire was not formally validated or psychometrically tested prior to administration.

The survey evaluation included an integrated analysis of demographic, medical and dental records and data collected from a 10-item IB questionnaire (supplemental material 1). The population consisted of a cohort of patients referred for periodontal reasons by general dental practitioners/other dental professionals to receive secondary care at the Periodontology Unit of the Guy’s and St Thomas’ Hospital, London (UK).

Eligible participants involved in King’s College London Oral, Dental and Craniofacial Biobank were included in this study (NHS UK Research Ethics Service approval reference 20/EE/0241, provided by the East of England-Cambridge East Research Ethics Committee). Each participant provided written consent to enrol in the Biobank. Approval to access data and samples for the present study was obtained from the Biobank Management Committee (REF027). The safety of participants’ data was maintained by following the current information governance regulations (GDPR). All participants’ data were anonymised, encrypted, and physically stored in a protected data point. The survey was conducted for 14 months, from November 2023 to January 2025. Referrals to secondary periodontal care were accepted according to the Unit’s referral criteria, which included patients with periodontitis (typically Stage III/IV, Grade B/C), as well as patients with other plaque-induced or non-plaque-induced periodontal diseases and mucogingival conditions, such as gingival recession.

The questionnaire was completed before the periodontal examination and before any oral hygiene instruction was provided by the specialist team. As per secondary care level, the following steps were followed: (1) the GDP’s written referral was reviewed before the periodontal examination including the reason for referral (periodontitis, other periodontal diseases), (2) collection of medical and dental history, (3) accurate patient-related periodontal history, periodontal risk factor assessment and oral hygiene habits (e.g. familiarity, frequence of toothbrushing, interdental brushing, etc.), (4) extra- and intra-oral examination (soft and hard tissues), (5) full mouth six-point pocket chart examination, (6) radiological examination (periapical x-rays, OPG, CBCT) according to clinical need, (7) anthropometric variables, including body weight, height, and waist circumference, were measured directly during the clinical visit with participants’ consent as part of the Biobank data collection protocol.

At the end of the periodontal examination, each case of periodontitis was accurately classified using the 2018 classification [10] (staging, grading and extension) by a consultant and UK specialist in periodontics. Clinical measurements were collected according to the Biobank standard operational protocol presented in a previous study [11].

### Questionnaire administration and analysis

A structured, self-administered questionnaire was developed to assess the awareness, usage patterns, perceptions, and barriers related to the use of IBs among participants enrolled in the Dental, Oral and Craniofacial Biobank. The questionnaire consisted of 10 items, primarily closed-ended, single-choice questions, with one optional open-ended question to capture additional qualitative feedback.

The questionnaire collected information across the following domains:

1. IB Usage Patterns.
2. Barriers and Motivating Factors.
3. Perceived Advantages and Drawbacks.
4. Financial Considerations.
5. Professional Recommendation and Clinical Background.
6. Comparison with Alternative Oral Hygiene Devices.
7. Qualitative Feedback: open-ended question.

Questions included a “Prefer not to say” option to minimise response bias and respect participant autonomy. The questionnaire was designed to be brief and easy to complete, taking approximately 5 min to finish.

### Sample size and data analysis

As no formal sample size calculation could be performed, due to the absence of previous similar studies, a convenience sample of 375 consecutive cases was included. In line with previous study methods [12], household income was collected indirectly by utilising area-level proxy derived using participants’ residential postcodes. These were mapped to the corresponding Lower Layer Super Output Areas (LSOAs), and mean household income estimates were obtained from publicly available UK Office for National Statistics (ONS) and related national statistics datasets.

### Data preprocessing and imputation

Participants who did not meet the 2018 case definition for periodontitis following specialist assessment were classified as the “Stage 0” group. This study-specific category included patients referred for suspected periodontal disease who were subsequently diagnosed with other periodontal or gingival conditions (e.g., gingivitis or incidental clinical/radiographic findings simulating periodontitis. Data cleaning and preprocessing were performed in Python (pandas and NumPy). All missing entries, including blank cells, non-standard missing values (e.g., “NA”, “nr”, “nan”, “–“), and responses of “Prefer not to say”, were coded as missing (NaN). Variables with more than 30% missing values were excluded from the analyses to minimise the risk of unreliable estimates resulting from extensive imputation. For the remaining variables, missing values were imputed according to variable type: continuous variables were imputed using the mean, ordinal variables using the median, and categorical variables using the mode (most frequent category). The cleaned dataset was subsequently exported to Excel for statistical analysis.

### Statistical analysis

Categorical variables were described using absolute frequencies and percentages. Frequencies participants of toothbrushing and continuous variables were summarised as mean ± standard deviation (SD), along with minimum–maximum ranges. Family income was additionally standardized (z-score) for multivariable modeling purposes. Unadjusted associations between daily use of IBs (daily vs. non-daily use) and selected socio-demographic and clinical variables were assessed using Pearson’s chi-square test. Specifically, associations were examined with periodontal stage (0–IV), periodontal grade (A–C).

To estimate independent determinants of daily IB use, penalised logistic regression models according to Firth were applied in order to reduce small-sample bias and potential quasi-complete separation due to imbalanced subgroup distributions. In the first model, severe periodontitis (stages III–IV vs. 0–II) was included as the primary predictor, adjusting for age (continuous), sex, and smoking status. In the second model, standardized family income (income_std) was analysed as the main predictor, adjusting for the same covariates. Results were reported as odds ratios (ORs) with 95% confidence intervals (95% CI). Penalised logistic regression according to Firth was also used to evaluate determinants of severe periodontitis (stages III–IV vs. 0/I/II) in the full sample and determinants of grade C periodontitis (vs. grades A/B) in complete cases. Covariates included standardized family income, age, sex, and smoking status.

## Results

The socio-demographic, behavioural, and clinical characteristics of the overall sample (*n* = 375) are reported in Table 1, questionnaires with more than 30% missing responses were excluded from the study (*n* = 57). The sample was predominantly female (60.0%). Regarding smoking habits, most participants reported never smoking (60.5%), whereas current smokers accounted for 8.8%. Vaping was reported in 9.9% of cases and alcohol consumption in 42.9%. Home oral-hygiene practices indicated a high brushing frequency, with 90.7% reporting brushing twice daily, and widespread use of an electric toothbrush (64.0%); daily mouthwash use was reported by 33.6%. Clinically, 86.7% of the sample had periodontitis; the stage distribution was markedly skewed toward advanced disease, with 49.9% classified as stage III and 24.8% as stage IV. Grade C was predominant (76.6%), and the localised form was most common (79.4%) (Table 1).

**Table 1:** Categorical variables of the study population. Distribution of socio-demographic, behavioral, and clinical categorical variables in the study sample. Values are presented as absolute frequency and percentage

Variable	Categories (frequency, %)
Gender	Female = 225 (60.0%); Male = 150 (40.0%)
Ethnicity	White = 223 (59.5%); Afro-Carabbean = 74 (19.7%); Asian = 54 (14.4%); other ethnicity Asian- White = 24 (6.4%).
Smoking history	Never = 227 (60.5%); Former = 115 (30.7%); Current = 33 (8.8%)
Vaping	No = 338 (90.1%); Yes = 37 (9.9%)
Alcohol use	No = 214 (57.1%); Yes = 161 (42.9%)
OHIP score	Low = 66 (17.6%); Moderate = 216 (57.6%); Severe = 78 (20.8%); Very severe = 15 (4.0%)
Toothbrush type	Manual = 91 (24.3%); Electric = 240 (64.0%); Both = 41 (10.9%); Other = 3 (0.8%)
Brushing frequency	Twice/day = 340 (90.7%); Once/day = 17 (4.5%); Three times = 13 (3.5%); Four times = 1 (0.3%); None = 4 (1.1%)
Mouthwash use	No = 249 (66.4%); Yes = 126 (33.6%)
Periodontitis stage	Stage 0 = 50 (13.3%); Stage I = 8 (2.1%); Stage II = 37 (9.9%); Stage III = 187 (49.9%); Stage IV = 93 (24.8%)
Periodontal status	Healthy = 38 (10.1%); Gingivitis = 12 (3.2%); Periodontitis = 325 (86.7%)
Periodontitis grade	A = 15 (4.6%); B = 61 (18.8%); C = 249 (76.6%);
Extent of periodontitis	Localised = 258 (79.4%); Generalised = 61 (18.8%); MIP = 6 (1.8%);

Stage 0 participants with no periodontitis. OHIP = Oral Health Impact Profile; MIP = molar-incisor pattern

Continuous variables are summarized in Table 2. Mean age was 50.45 ± 15.4 years (range 16–88). Mean household income was £36,684 ± 3,789, with values ranging from £29,000 to £47,000. With respect to behavioural variables, the mean cigarette consumption was 3.72 ± 6.78/day and the mean smoking duration was 7.12 ± 11.36 years; among former smokers, years since cessation averaged 4.93 ± 10.28. Mean alcohol intake was 3.31 ± 5.93 units/week, whereas vaping duration was overall limited (0.26 ± 1.17 years). Anthropometric parameters indicated a mean height of 168.1 ± 9.4 cm, mean weight of 74.55 ± 17.6 kg, and mean waist circumference of 91.4 ± 14.9 cm. In terms of dental variables, the mean number of teeth present was 26.1 ± 4.3 (24.8 ± 4.02 excluding third molars), while the mean number of implants was low (0.13 ± 0.80), with a maximum observed value of 8 (Table 2).

**Table 2:** Descriptive statistics showing continuous variables of the study population

Variable	Mean ± Standard Deviation	Min–Max
Household income (£)	36,684 ± 3,789	29,000–47,000
Cigarettes/day	3.72 ± 6.78	0–60
Smoking years	7.12 ± 11.36	0–50
Years since quitting	4.93 ± 10.28	0–60
Alcohol units/week	3.31 ± 5.93	0–50
Height (cm)	168.1 ± 9.4	147–194.5
Weight (kg)	74.55 ± 17.6	38–160
Waist circumference (cm)	91.4 ± 14.9	34–144
Age (years)	50.45 ± 15.4	16–88
Vaping duration (years)	0.26 ± 1.17	0–10
Number of teeth	26.1 ± 4.3	5–32
Number of teeth (excluding 8 s)	24.8 ± 4.02	5–31
Number of implants	0.13 ± 0.80	0–8

“Number of teeth (excluding 8 s)” refers to the number of teeth excluding third molars

### Use of IB and periodontal staging and grading

**Fig. 1:**
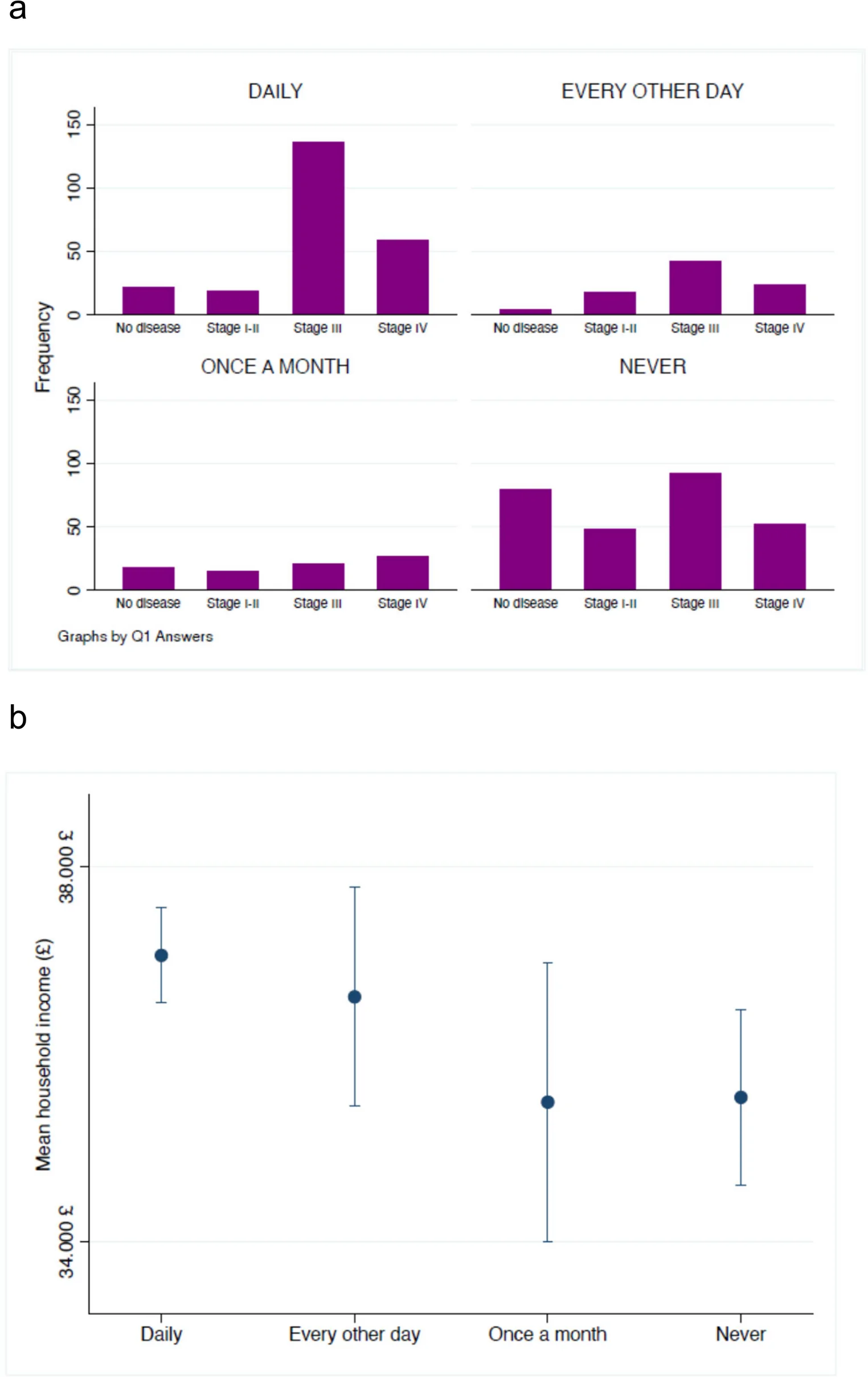
**a** Predicted probability of daily use of interdental brushes by periodontitis stage 1. **b** Predicted probability of daily use of interdental brushes by income level

Figure 1.a shows predicted probability of daily use of IBs by periodontitis stage. Unadjusted bivariate analyses between daily IB use and clinical variables showed a significantly association with periodontal stage (χ² = 41.91; *p* < 0.001) but not with periodontal grade (χ² = 2.68; *p* = 0.848). Results from Firth penalized multivariable models assessing determinants of daily IB use are reported in Table 3A–B. In the model where severe periodontitis (stages III–IV) was the main predictor (Table 3A), participants with severe periodontitis (Stages III–IV) had more than three times the odds of reporting daily IB use compared with participants in the combined Stage 0–II group (OR = 3.11; 95% CI 1.88–5.15; *p* < 0.001), after adjustment for age, sex, and smoking status.

**Table 3:** Firth penalised logistic regression for daily interdental brush use. Panel A reports the association between daily interdental brush use and periodontitis severity (Stage III–IV vs non-severe), adjusted for age, sex, and smoking. Severe periodontitis and increasing age were significantly associated with daily use, whereas current smoking was inversely associated. Panel B shows the association between daily use and standardized income (income_std), adjusted for the same covariates. Higher income and older age were positively associated with daily use, while current smoking remained negatively associated. Values are reported as odds ratios (OR) with 95% confidence intervals (CI). Stage 0 represents participants who did not meet the 2018 case definition for periodontitis following specialist assessment (study-specific comparator group). Reference categories were non-severe periodontitis (Stage 0–II), female sex, and never smokers. *p < 0.05; **p < 0.01; ***p < 0.001

A)		
Variable	Odds Ratio (95% CI)	*p*-value
Severe periodontitis (III–IV vs. non-severe)	3.11 (1.88–5.15)	< 0.001***
Age (per year increase)	1.02 (1.00–1.03)	0.018*
Former smoker (vs. never)	1.61 (0.95–2.71)	0.076
Current smoker (vs. never)	0.31 (0.15–0.67)	0.003**
Male (vs. female)	0.79 (0.50–1.25)	0.313
B)		
Variable	Odds Ratio (95% CI)	*p*-value
Standardized household income (z-score)	1.38 (1.08-1.77)	0.010*
Male	0.91(0.57-1.42)	0.676
Former smoker	1.61 (0.98-2.75)	0.073
Current smoker	0.41 (0.18-0.87)	0.021*
Age	1.024 (1-1.04)	0.002**

### Use of IB and mean household income

Figure 1.b shows predicted probability of daily use of IBs by income level. A one-way ANOVA was conducted to assess differences in mean household income across the Q1 groups (Daily, Every other day, Once a month, Never), with Bonferroni correction applied for multiple comparisons. In the sample (*N* = 375), the overall mean household income was £36,684.26 (SD = £3,789.48). The highest mean values were observed in the Daily group, whereas the lowest were found in the Once a month and Never groups. The ANOVA revealed a statistically significant difference among groups, F(3, 371) = 3.41, *p* = 0.0178. Bartlett’s test was not significant, supporting the assumption of homogeneity of variances. Post hoc comparisons using the Bonferroni adjustment indicated a significant difference only between the Daily and Never groups, with higher mean income in the Daily group. No other pairwise comparisons reached statistical significance.

Family income was also computed as categorical variables into tertiles (≤ £35,000; £35,001–37,000; > £37,000) and use as main predictors assessing determinants of daily interdental (Table 3.b). Income_std was independently associated with higher odds of daily use (OR = 1.38; 95% CI 1.08–1.77; *p* = 0.010). Finally, when adjusted for disease severity as outcomes only in daily IBs users, income was not associated with disease severity (Table 4).

**Table 4:** Associations between daily interdental brush use, severe periodontitis (III–IV vs. 0/I/II) as outcome with socio-demographic and behavioural variables

Variable	OR (95% CI)	*p*-value
Standardized household income (z-score)	1.04 (0.74–1.47)	0.791
Age (per year)	1.02 (0.99–1.04)	0.172
Former smoker (vs. never)	1.06 (0.52–2.18)	0.853
Current smoker (vs. never)	1.82 (0.32–10.51)	0.498
Male (vs. female)	1.39 (0.67–2.86)	0.365

### Use of IB and age and smoking status

In both logistic regression models, age was positively associated with daily use of IBs (Table 3.a: OR = 1.02, 95% CI = 1–1.03, *p* = 0.018; Table 3.b: OR = 1.024 1–1.04 *p* = 0.002), whereas current smoking showed a strong inverse association (OR = 0.31, 95% (0.15–0.67), *p* = 0.003: Table 3.b: OR = 0.41, 95% CI = 0.18–0.87, *p* = 0.021). Former smoking and gender were not significantly related to daily use not even when adjusted for disease severity (Table 4).

**Fig. 2:**
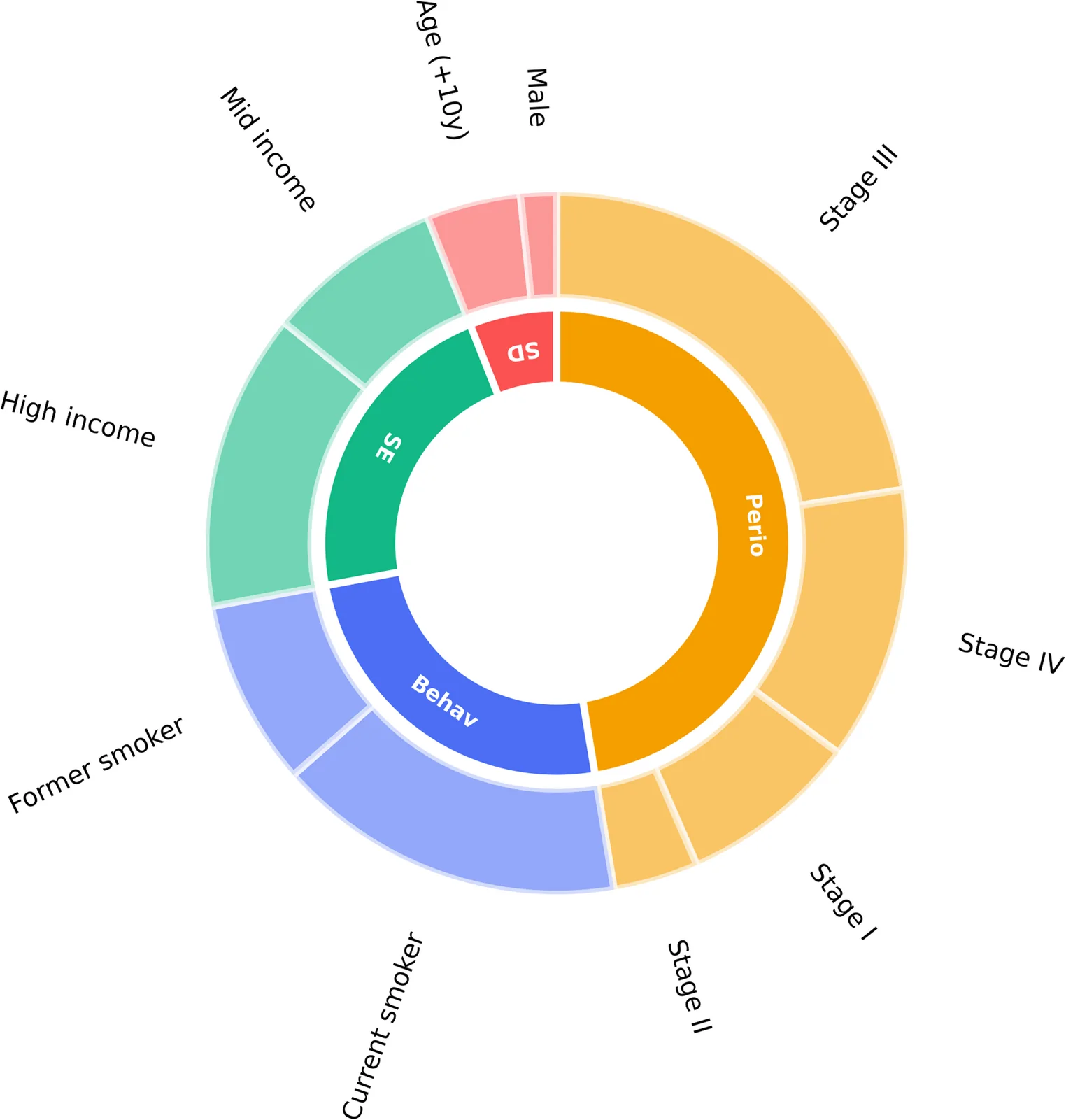
Relative importance of predictors of daily interdental brush use Relative feature importance's. The features with the highest relative importance’s are stage III (22.5%), current smoker (16.1%), High Income (13.7%), and Stage IV (12.6). Perio: Periodontitis, SD: Socio-Demographic, SE: Socio Economic

Figure 2 summarises the relative contribution of key predictor domains to daily IB use. Sector sizes are proportional to the absolute magnitude of the log-odds coefficients (|log(OR)|) derived from multivariable logistic regression models. The periodontitis stages (I–IV) were obtained from the stage-adjusted model (Table 4.A), whereas socioeconomic and behavioural predictors (household income, smoking status, age, and sex) were derived from the income-adjusted model (Table 4.B). Larger sectors indicate stronger relative associations with daily IB use.

### Use of IB and questionnaire

Of the total sample, 236 participants reported daily IB use (Q1). Among non-daily users, the most commonly reported reason for non-use was difficulty finding the correct brush size (32%) (Q2). Most non-daily users (71%) indicated that cost was not a motivating factor (Q3). Among daily IB users (*n* = 236), the most commonly reported IB replacement frequency (Q4) was once per week, reported by 54.2% of participants (*n* = 128). The most commonly perceived drawback of IBs (Q5) was difficulty cleaning back teeth (38%), followed by the need to use multiple sizes/colours (28%) and concerns about gaps increasing between teeth (22%).Regarding the professional recommendation of IBs(Q7), IB use was most frequently first recommended by a general dentist, as reported by 63.6% of respondents (*n* = 150). Regarding perceived equivalence with waterflosser (Q9), the majority of participants (57.6%, *n* = 136) reported being unfamiliar with waterfloss devices. Among those who were familiar (43.4% *n* = 100), 25.0% (*n* = 59) indicated a preference for IBs over waterflossers. A smaller proportion of daily users (8.9%, *n* = 21) reported feeling that the waterflosser could perform the same function as IBs, while 8.5% (*n* = 20) reported using both devices.

#### Question 10: patient's reported feedback about the use of IB

Out of 38 feedback comments analysed, 84% of participants reported problems or negative experiences associated with IB use, while 13.2% of participants expressed positive experiences or satisfaction with their effectiveness; one comment was neutral or unclear. Negative comments commonly related to pain, gingival bleeding, and discomfort, particularly from the metal wire. Patients frequently reported brushes bending or breaking easily, difficulty accessing posterior teeth, and the need for multiple brush sizes. Other concerns included perceived widening of interdental spaces, high cost, environmental impact, and time required for use. Few patients preferred alternatives such as dental floss, water flossers, or toothpicks due to greater comfort and convenience.

Supplemental material 2 reports analyses used chi-square tests, ordinal logistic regression, bivariate analyses, and Firth penalized logistic regression to examine associations between questionnaire items and periodontal or participant characteristics.

## Discussion

This study investigated patterns of IB use among patients attending secondary periodontal care and explored how disease severity, socioeconomic factors, age and smoking status relate to interdental cleaning behaviour. The findings demonstrate that IB use is strongly associated with clinical severity of periodontitis and social determinants, particularly income and smoking status.

A key finding of this study is that patients with more advanced periodontitis, particularly Stage III disease, were significantly more likely to report daily IB use. This may be in line with previous finding that demonstrated that patients with severe stages of periodontitis are more aware of signs and symptoms and of risk of tooth loss, which can justify the higher adherence to the daily use of IB compared to milder stages of disease. Santamaria et al., [13]. In contrast, individuals without periodontitis showed the lowest prevalence of interdental cleaning, in agreement with previous results that showed how the dimension of interdental space can affect the regular use of IBs in individuals without periodontitis [14].

However, the observed association between more advanced periodontitis and daily IB use should be interpreted cautiously. Given the cross-sectional design, it is not possible to establish when participants initiated interdental brushing or whether its adoption occurred before or after periodontal disease progression. Furthermore, as participants were recruited from a secondary periodontal care referral population, those with more advanced disease may have been more likely to have previously received professional oral hygiene advice. This interpretation is supported by our finding that most participants reported first receiving this recommendation from their general dentist.

This association can be also related to anatomical changes that occur with the onset and progression of periodontitis [14]. In particular, as periodontal destruction advances, interdental spaces enlarge, making IBs easier to insert and more effective than floss. This may enhance physical capability and perceived usefulness, both central constructs in the COM-B model. At earlier stages of disease, when interdental spaces are narrower and symptoms minimal, patients may perceive IBs as unnecessary, uncomfortable, or difficult to use, limiting uptake [14]. These findings further emphasise that effective self-performed plaque control is fundamental not only for preventing periodontal disease but also for achieving favourable treatment outcomes and maintaining periodontal stability following therapy [1].

Current smoking emerged as one of the strongest negative predictors of daily IB use and was also associated with more severe periodontal disease. This dual effect highlights smoking as a critical behavioural and clinical risk factor. Smokers may experience reduced gingival bleeding, which can mask disease severity and reduce perceived need for interdental cleaning [15]. Additionally, smoking is associated with lower health motivation, reduced engagement with preventive behaviours, and weaker adherence to professional advice [16]. These findings underscore the need for tailored behaviour change interventions that integrate smoking cessation support with oral hygiene instruction, rather than addressing these behaviours in isolation.

This study also identified a clear socioeconomic gradient in IB use, with participants from higher-income areas significantly more likely to report daily use, confirming similar findings about flossing reported in a previous study [17]. Importantly, our study showed that household income was not associated with periodontal stage or grade, suggesting that differences in IB use are not driven by disease severity alone, but by broader social and environmental conditions.

Lower-income individuals may face reduced physical opportunity for interdental cleaning, including financial constraints, limited access to oral hygiene products, and competing life priorities. Perceived cost was a commonly reported barrier, consistent with previous studies showing that preventive oral health behaviours cluster with socioeconomic advantage [18]. These findings highlight that knowledge alone is insufficient to promote behaviour change and that structural barriers must be addressed. Interventions such as subsidised IBs, inclusion of IBs in NHS preventive care packages, or provision of starter kits during periodontal appointments may help reduce inequalities in oral hygiene behaviours.

Older age was modestly but consistently associated with more frequent IB use. While age was also associated with more severe periodontal stages, older adults appeared more willing or able to adopt interdental cleaning behaviours, possibly due to higher perceived vulnerability and stronger health-related routines [19]. These findings suggest that younger patients represent a key target group for early preventive interventions, particularly before irreversible periodontal damage occurs [20].

Additional findings from the questionnaire indicate that while IBs are widely recommended and used on a daily basis, their long-term acceptability remains limited for a substantial proportion of users. This is in line with a previous investigation that reported more discomfort and less compliance for patent using metal wire IB compared to rubber interdental picks [21].

Most participants reported received initial recommendations from general dentists, qualitative feedback revealed considerable dissatisfaction with their use. Practical concerns such as the need for multiple brush sizes, perceived widening of interdental spaces, cost, and environmental impact should be taken into consideration by dental professionals. Where such concerns may negatively affect patient compliance with daily interdental cleaning, alternative devices should be considered.

The strengths of this study include a well-represented clinical sample, use of the 2018 periodontal classification by specialist clinicians, and integration of behavioural and socioeconomic data. Several limitations should be acknowledged. First, the questionnaire was developed specifically for this study and was not formally validated or psychometrically tested. Although the items were informed by previous literature and clinical experience, measurement error cannot be excluded. In addition, some response categories, particularly those assessing IB frequency, were not equally spaced (e.g., “every other day” versus “once a month”), which may have limited the ability to discriminate between different levels of behavioural adherence. Consequently, the questionnaire should be considered exploratory, and future studies should employ validated instruments with more granular response options to improve reliability, sensitivity and comparability across populations. As the study relied on a self-administered questionnaire, social desirability and response bias cannot be excluded. Participants may have over-reported favourable oral hygiene behaviours, meaning that self-reported interdental brush use may not fully reflect actual practice. An additional limitation relates to the household income that was estimated using mean income values for the residential area derived from postcode-linked national statistics. Although area-level measures provide a pragmatic proxy for socioeconomic circumstances, they may not accurately reflect the actual income or socioeconomic position of individual participants. Finally, the study population was drawn from a secondary care setting, limiting generalisability to primary care or community samples.

In conclusion, our findings identify a distinct profile of periodontal patients who are less likely to use IBs regularly, characterised by early stages of periodontitis, younger age, lower income, and current smoking status. Common dissatisfaction related to practical, financial, and environmental concerns underscores the need for tailored preventive advice and consideration of personalised interdental cleaning strategies to improve adherence.

## Supplementary Information

Below is the link to the electronic supplementary material.

Supplementary File 1 (DOCX 60.3 KB)

Supplementary File 2 (DOCX 26.9 KB)

## Data Availability

No datasets were generated or analysed during the current study.
