# Supplementary material for: Interdental brushing behaviour in adults: Influence of periodontal status and socioeconomic factors
Source: Clin Oral Investig. 2026 Sep 16;30(10):447. doi: 10.1007/s00784-026-07129-7 (PMC13582308; doi:10.1007/s00784-026-07129-7)
Supplement: Supplementary file 1 [file 784_2026_7129_MOESM1_ESM.docx]

Version 1.0, 04 October 2023

PERIO 10-009

IRAS project ID:255079

Participant Biobank Number _____________

**INTERDENTAL BRUSHES SURVEY for participants in Dental, Oral and Craniofacial Biobank**

Please choose only one option

1. How often do you use interdental brushes?
A. At least once a day.
B. Every other day.
C. Once a month.
D. Never.
E. Prefer not to say.
2. If you are not using them every day, what is the reason?
A. Interdental brushes are too expensive in relation to their duration.

B. I find difficult to find the correct size for my teeth.

C. I think I can clean properly my teeth with just the toothbrush.

D. I prefer to floss my teeth.

E. I prefer to use the waterfloss.

F. Prefer not to say, other, never heard of them .

3. If you are not using them every day, would you be more motivated in using interdental brushes if they were cheaper?
A. Yes, I think the price is the reason why I’m not using Interdental brushes.
B. No, I don’t think the price is the reason why I am not using them.
4. How often do you throw the Interdental brushes after the use?
A. Every day.
B. Every 3 days.
C. Every week.
D. Prefer not to say.
E. More than 1 week
5. What do you think is the major drawback of Interdental brushes?
A. I struggle to clean my back teeth.
B. They are making the gaps between my teeth bigger than before.
C. I need to use too many colours and size.
D. Plastic Interdental brushes are not recyclable.
E. other
6. How much do you pay for a small bag of Interdental brushes (8 pieces)
A. Less than £2.
B. Between £2.00 to £6.00.
C. More than £6.00.
D. I do not know.
E. Prefer not to say.
7. Who was the first to recommend you to the use of Interdental brushes?
A. The General Dentist
B. The Hygienist
C. The Periodontist
D. Others
8. How long have you been told you have gum disease?
A. Less than 1 year.
B. Between 1 year and 5 years.
C. Between 5 years and 10 years
D. More than 10 years.
E. I do not have gum disease
9. Do you think the waterfloss is able to do the same job of interdental brushes?
A. Yes, I have this feeling.
B. No, I prefer to use interdental brushes.
C. I use both.
D. I do not know what a waterfloss is.
10. Do you have any comments about the use of Interdental brushes?

________________________________________________________________________________________________________________________________________________
