# Supplementary material for: Interdental brushing behaviour in adults: Influence of periodontal status and socioeconomic factors
Source: Clin Oral Investig. 2026 Sep 16;30(10):447. doi: 10.1007/s00784-026-07129-7 (PMC13582308; doi:10.1007/s00784-026-07129-7)
Supplement: Supplementary file 2 [file 784_2026_7129_MOESM2_ESM.docx]

Supplementary exploratory analyses were conducted to further investigate associations between questionnaire items (Q1–Q9) and periodontal stage or grade using chi-square tests. Predicted probabilities of belonging to each periodontal stage were estimated using ordinal logistic regression models stratified by income tertiles, with marginal probabilities calculated post-estimation. Within the non-daily users subgroup, additional bivariate analyses were performed between Q2 and demographic, behavioural, and periodontal variables. Finally, among non-daily interdental brush users with a valid Q3 response, the association between Q3 (willingness to use interdental brushes if cost were lower; yes vs no) and demographic, socio-economic, behavioural, and periodontal variables was assessed using Firth penalized logistic regression, given the limited number of events in some categories.. For multi-level periodontal variables, dummy coding was applied with global Wald tests. All statistical tests were two-sided, and statistical significance was set at p < 0.05.

## **Table S1. Chi-squared associations between questionnaire items (Q1–Q9) and periodontitis stage. (supplementary).**

| **Question** | **Chi-squared** | **p-value** |
| --- | --- | --- |
| Q1 | 41,9 | <0.001 |
| Q4 | - | 0.536 |
| Q6 | - | 0.132 |
| Q7 | 40,3 | 0.001 |
| Q8 | 36,1 | 0.015 |
| Q9 | - | 0.385 |

This table reports the χ² values and corresponding p-values from bivariate analyses assessing the association of individual questionnaire items with periodontitis stage.

## **Table S2. Chi-squared associations between questionnaire items (Q1–Q9) and periodontitis grade. (supplementary).**

| **Question** | **Chi-squared** | **p-value** |
| --- | --- | --- |
| Q1 | - | 0.848 |
| Q4 | - | 0.405 |
| Q6 | - | 0.481 |
| Q7 | - | 0.375 |
| Q8 | - | 0.371 |
| Q9 | - | 0.829 |

This table reports the χ² values and corresponding p-values from bivariate analyses assessing the association of individual questionnaire items with periodontitis grade.

## **Table S3. Predicted probabilities of periodontitis stage (I–IV) by income terciles (supplementary).**

| **Group** | **Predicted probability** |
| --- | --- |
| Stage I, Income Low | 2.2% |
| Stage I, Income Mid | 2.7% |
| Stage I, Income High | 2.5% |
| Stage II, Income Low | 10.3% |
| Stage II, Income Mid | 12.5% |
| Stage II, Income High | 11.7% |
| Stage III, Income Low | 56.6% |
| Stage III, Income Mid | 58.5% |
| Stage III, Income High | 57.9% |
| Stage IV, Income Low | 30.9% |
| Stage IV, Income Mid | 26.3% |
| Stage IV, Income High | 27.9% |

Predicted probabilities of belonging to each periodontitis stage (I–IV), stratified by household income terciles (Low income: ≤ £35,000; Middle income: £35,001–£37,000; High income: > £37,000), estimated from an ordinal logistic regression model restricted to participants with periodontitis (n = 325). Predicted probabilities were obtained using post-estimation marginal predictions.

**Table S4. Bivariate analyses between Q2 categories and demographic, behavioral and periodontal variables**

| **Variable tested** | **Type of variable** | **Statistical test** | **Test statistic** | **p-value ((p<0.05)** |
| --- | --- | --- | --- | --- |
| Gender | Categorical (2 levels) | Pearson χ² + Fisher | χ²(5)=10.80 | 0.056 (Fisher=0.050) |
| Smoking history | Categorical (3 levels) | Pearson χ² + Fisher | χ²(10)=12.47 | 0.255 (Fisher=0.319) |
| OHIP score | Categorical (4 levels) | Pearson χ² | χ²(15)=16.79 | 0.332 |
| Periodontal stage | Categorical (5 levels) | Pearson χ² | χ²(20)=39.31 | 0.006 ** |
| Periodontitis grade | Categorical (3 levels) | Pearson χ² + Fisher | χ²(10)=12.16 | 0.275 (Fisher=0.241) |
| Periodontitis extent | Categorical (3 levels) | Pearson χ² + Fisher | χ²(10)=14.68 | 0.144 (Fisher=0.140) |
| Periodontitis presence (Stage 0 vs Stages I–IV) | Categorical (2 levels) | Pearson χ² + Fisher | χ²(5)=6.57 | 0.255 (Fisher=0.260) |

**Table S4** reports the bivariate analyses assessing the association between Q2 response categories and selected demographic, behavioural, and periodontal variables using Pearson’s chi-square test and Fisher’s exact test where appropriate.

**Table S5. Univariate Firth Penalized Logistic Regression Analyses for Q3 (Motivation to Use Interdental Brushes if Cheaper)**

**A)**

| **Predictor** | **OR (95% CI)** | **p-value** |
| --- | --- | --- |
| Age | 0.99 (0.97–1.01) | 0.320 |
| Weight (kg) | 1.01 (0.99–1.02) | 0.535 |
| Waist circumference | 1.01 (0.99–1.04) | 0.239 |
| Height (cm) | 0.98 (0.96–1.01) | 0.176 |
| Vaping (yes vs no) | 1.26 (0.42–3.75) | 0.683 |
| Vaping duration (years) | 1.06 (0.82–1.37) | 0.650 |
| Smoking years | 0.97 (0.93–1.01) | 0.130 |
| Smoking intensity | 0.98 (0.91–1.07) | 0.701 |
| Smoking years since quitting | 1.00 (0.95–1.05) | 0.884 |
| Gender (male vs female) | 0.76 (0.34–1.72) | 0.510 |
| Periodontitis (Stages I–IV vs Stage 0) | 1.55 (0.55–4.36) | 0.409 |
| OHIP score | 1.27 (0.73–2.21) | 0.403 |
| Number of teeth | 1.03 (0.96–1.11) | 0.425 |
| Number of implants | 0.96 (0.19–4.81) | 0.959 |
| Mouthwash daily use | 0.68 (0.27–1.72) | 0.414 |
| Alcohol units/week | 0.98 (0.90–1.08) | 0.716 |
| Mean household income | 1.00 (0.9999–1.0001) | 0.856 |
| Income (standardized) | 1.04 (0.66–1.64) | 0.856 |

**B) Periodontitis Stage (reference = Stage 0)**

| **Predictor** | **OR (95% CI)** | **p-value** |
| --- | --- | --- |
| Stage I vs 0 | 1.68 (0.20–14.09) | 0.635 |
| Stage II vs 0 | 1.13 (0.28–4.63) | 0.860 |
| Stage III vs 0 | 1.16 (0.36–3.70) | 0.807 |
| Stage IV vs 0 | 2.96 (0.90–9.74) | 0.074 |
| **Global Wald test** | — | **0.304** |

**C) Periodontitis Grade (reference = Grade A)**

| **Predictor** | **OR (95% CI)** | **p-value** |
| --- | --- | --- |
| Grade B vs A | 0.90 (0.04–22.97) | 0.948 |
| Grade C vs A | 3.76 (0.19–75.63) | 0.387 |
| **Global Wald test** | — | **0.101** |

**D) Periodontitis Extent (reference = Localized)**

| **Predictor** | **OR (95% CI)** | **p-value** |
| --- | --- | --- |
| Generalized vs Localized | 0.68 (0.19–2.45) | 0.553 |
| MIP vs Localized | 1.73 (0.32–9.43) | 0.526 |
| **Global Wald test** | — | **0.655** |

**Table S5** presents univariate Firth penalized logistic regression analyses examining the association between Q3 (motivation to use interdental brushes if cheaper; Yes vs No) and demographic, behavioural, socioeconomic, and periodontal variables among non-daily interdental brush users with a valid Q3 response. Panel A reports associations with continuous and binary predictors. Panels B, C, and D report category-specific odds ratios for periodontal stage, grade, and extent, respectively, using the specified reference categories and including global Wald tests for the multi-level variables. Odds ratios (ORs) with 95% confidence intervals (CIs) are reported. Firth penalized logistic regression was used to reduce small-sample bias and address potential quasi-complete separation due to sparse data.

**Table S6. Association Between Q2 Response Categories and Gender (Chi-square and Fisher’s Exact Tests)**

| **Q2 Reason** | **Female n (%)** | **Male n (%)** | **Total n (%)** |
| --- | --- | --- | --- |
| A | 6 (46.15%) | 7 (53.85%) | 13 (10.74%) |
| B | 21 (77.78%) | 6 (22.22%) | 27 (22.31%) |
| C | 17 (70.83%) | 7 (29.17%) | 24 (19.83%) |
| D | 15 (57.69%) | 11 (42.31%) | 26 (21.49%) |
| E | 3 (60.00%) | 2 (40.00%) | 5 (4.13%) |
| F | 10 (38.46%) | 16 (61.54%) | 26 (21.49%) |
| Total | 72 (59.50%) | 49 (40.50%) | 121 (100%) |

**χ²(5) = 10.80, p = 0.056; Fisher’s exact p = 0.050**

**Table S6.** Distribution of Q2 response categories according to gender (female vs male) among non-daily interdental brush users. Associations were assessed using Pearson’s chi-square test; Fisher’s exact test was applied due to small expected cell counts

**Table S7. Association Between Q2 Response Categories and Periodontitis Stage (Chi-square Tests)**

| **Q2 Reason** | **No disease n (%)** | **Stage I n (%)** | **Stage II n (%)** | **Stage III n (%)** | **Stage IV n (%)** | **Total n (%)** |
| --- | --- | --- | --- | --- | --- | --- |
| A | 1 (7.69%) | 0 (0.00%) | 1 (7.69%) | 6 (46.15%) | 5 (38.46%) | 13 (10.74%) |
| B | 4 (14.81%) | 0 (0.00%) | 6 (22.22%) | 13 (48.15%) | 4 (14.81%) | 27 (22.31%) |
| C | 5 (20.83%) | 1 (4.17%) | 8 (33.33%) | 1 (4.17%) | 9 (37.50%) | 24 (19.83%) |
| D | 9 (34.62%) | 2 (7.69%) | 2 (7.69%) | 9 (34.62%) | 4 (15.38%) | 26 (21.49%) |
| E | 2 (40.00%) | 1 (20.00%) | 0 (0.00%) | 1 (20.00%) | 1 (20.00%) | 5 (4.13%) |
| F | 4 (15.38%) | 0 (0.00%) | 1 (3.85%) | 14 (53.85%) | 7 (26.92%) | 26 (21.49%) |
| Total | 25 (20.66%) | 4 (3.31%) | 18 (14.88%) | 44 (36.36%) | 30 (24.79%) | 121 (100%) |

**χ²(20) = 39.31, p = 0.006**

**Table S7.** Distribution of Q2 response categories across periodontal stages (no disease, Stage I–IV) among non-daily interdental brush users (n = 121). Associations were evaluated using Pearson’s chi-square test.

**Table S8. Association Between Q2 Response Categories and Presence of Periodontitis (Chi-square and Fisher’s Exact Tests)**

| **Q2 Reason** | **No periodontitis n (%)** | **Periodontitis n (%)** | **Total n (%)** |
| --- | --- | --- | --- |
| A | 1 (7.69%) | 12 (92.31%) | 13 (10.74%) |
| B | 4 (14.81%) | 23 (85.19%) | 27 (22.31%) |
| C | 5 (20.83%) | 19 (79.17%) | 24 (19.83%) |
| D | 9 (34.62%) | 17 (65.38%) | 26 (21.49%) |
| E | 2 (40.00%) | 3 (60.00%) | 5 (4.13%) |
| F | 4 (15.38%) | 22 (84.62%) | 26 (21.49%) |
| **Total** | **25 (20.66%)** | **96 (79.34%)** | **121 (100%)** |

**χ²(5) = 6.57, p = 0.255; Fisher’s exact p = 0.260**

**Table S8.** Distribution of Q2 response categories according to periodontitis status (Stage 0 vs Stages I–IV) among non-daily interdental brush users (n = 121). Associations were assessed using Pearson’s chi-square test and Fisher’s exact test when appropriate.
